# Bisphosphonates for the Treatment of Calcinosis Cutis—A Retrospective Single-Center Study

**DOI:** 10.3390/biomedicines9111698

**Published:** 2021-11-16

**Authors:** Lilian Rauch, Rüdiger Hein, Tilo Biedermann, Kilian Eyerich, Felix Lauffer

**Affiliations:** 1 Department of Dermatology and Allergy, Technical University of Munich, 80802 Munich, Germany; lilianrauch@t-online.de (L.R.); ruediger.hein@mri.tum.de (R.H.); tilo.biedermann@tum.de (T.B.); kilian.eyerich@tum.de (K.E.); 2 Center for Molecular Medicine, Division of Dermatology and Venereology, Department of Medicine Solna, Karolinska Institute, 17177 Stockholm, Sweden

**Keywords:** calcinosis cutis, bisphosphonates, pamidronate, autoimmune connective tissue diseases, systemic sclerosis, dermatomyositis, treatment

## Abstract

(1) Background: Calcinosis cutis is a frequent symptom of autoimmune connective tissue diseases leading to pain, transcutaneous expulsion of calcified material and bacterial superinfection. There is a high need for new therapeutic options as no standardized treatment algorithm is established. While case reports indicate beneficial effects of bisphosphonates, standardized evaluation of treatment effects is missing. (2) Methods: In this retrospective analysis we evaluate the effects of intravenous pamidronate, a second-generation bisphosphonate, in seven patients with calcinosis cutis using consecutive clinical pictures, radiological examinations and patient’s subjective evaluation. (3) Results: 5/6 patients reported a reduction of pain, improvement of general condition and cessation of calcinosis progression. Regression of skin lesions was detectable in clinical pictures of 2/6 patients, while 1/6 patients had stable disease. Radiological examination revealed improvement or stable disease in 3/5 patients. Fever was the most common side effect. One out of seven patients developed osteonecrosis of the jaw. (4) Conclusions: Bisphosphonates appear to have beneficial effects in a subgroup of calcinosis cutis patients. While patient’s subjective evaluation was mainly positive, objective assessments showed improvement in approximately half of the cases. With regard to potential severe side effects, a careful risk-benefit evaluation is necessary before treatment initiation.

## 1. Introduction

Calcinosis cutis (CC) is a frequent symptom of autoimmune connective tissue diseases, which severely impacts patient’s quality of life. It is defined as a deposition of insoluble calcium in the subcutaneous tissue, while serum calcium and/or phosphate levels are not elevated in most cases [1]. CC forms subcutaneous nodules, which are usually localized at mechanically stressed body sites, such as fingers or above joints. Often it causes inflammation and devastating perforations of the skin, leading to pain and baring the risk of bacterial superinfection. CC can occur in all autoimmune connective tissue diseases, but is most frequently seen in patients suffering from dermatomyositis and systemic sclerosis [2]. The pathogenesis is so far poorly understood. Chronic hypoxia, local trauma and inflammation are potential factors favouring the de novo development and maintenance of CC. Known associations to CC are female sex, chronic disease course, osteoporosis, digital ulcers and anti-centromere autoantibodies [3]. The treatment of CC is challenging as no standardized treatment guidelines have been established. Most recommendations are based on case reports or small case series, while randomized controlled trials are missing. Published treatment attempts include intralesional corticosteroid injections, topical or intravenous thiosulfate, warfarin, minocycline, colchicine, calcium channel blockers, intravenous immunoglobulins, surgical excision and ablative LASER therapy [4]. However, variability of individual response to therapy is high and additional therapeutic concepts are needed.

Pamidronate is a second-generation disodium bisphosphonate, which is approved for the treatment of hypercalcemia of malignancy, osteolytic bone metastases of breast cancer and multiple myeloma, as well as Paget disease of the bone. Second-generation bisphosphonates inhibit farnesyl pyrophosphate synthase, a key enzyme of the mevalonate pathway. The inhibition leads to a reduction of posttranslational modifications of guanosine triphosphate binding proteins, which are essential for the activity of osteoclasts [5]. Based on this mode of action, bisphosphonates prevent atrophy of bone tissue and lead to a decrease of calcium serum levels. Stabilization and/or improvement of CC was observed in two case reports [6,7]. However, a case series of six patients showed no beneficial effects of etidronate on clinical and radiological assessments of CC in six patients with dermatomyositis and systemic sclerosis [8].

In this retrospective study we evaluate the effects of intravenous pamidronate in seven patients with dermatomyositis, systemic sclerosis or mixed connective tissue diseases. We assessed clinical outcomes based on photo documentation and radiological examination, as well as subjective therapy-related benefits perceived by the patients using questionnaires.

## 2. Materials and Methods

Seven patients diagnosed with “calcinosis cutis” who received treatment with pamidronate at our department between 2010 and 2020 were identified. This comprises all cases of severe CC in our department during this period of time. Of those, 2 patients died before the beginning of the study. Clinical information was assessed by clinical records, radiological reports and photography taken during hospital stays. Clinical and radiological improvement were always evaluated in the last photograph or radiological examination, which was available. The median time between the first pamidronate infusion and the last photograph for clinical assessment was 41.5 (9–66) months. The median time between the first pamidronate infusion and the last radiologic assessment was 19 (11–37) months. Five patients filled out a questionnaire (see Supplementary Materials) about personal perceptions of bisphosphonate therapy effects. The median time between the last pamidronate infusion and the completion of the questionnaire (follow up) was 30 (15–98) months. The full questionnaire can be found in Supplementary Material S1. Data was visualized using GraphPad Prism 7 software (San Diego, CA, USA). The study protocol was approved by the Ethics Committee of the School of Medicine, Technical University of Munich (321/20) and written informed consent was obtained from all patients.

## 3. Results

### 3.1. Patient’s Characteristics

Between 2010 and 2020 six female and one male patient received 70–75 mg of intravenous pamidronate every 12 weeks for three consecutive days for the treatment of CC at our department (Table 1). Underlying autoimmune diseases were dermatomyositis (*n* = 2), mixed connective tissue diseases (*n* = 3) and systemic sclerosis (*n* = 2). The time between the onset of CC and the initiation of the pamidronate treatment was 7 ± 4.2 years. Six out of seven patients received immunosuppressive co-medication before and during pamidronate treatment, mostly with prednisolone, methotrexate or intravenous immunoglobulins. The number of bisphosphonate cycles per patient was 8.6 ± 5.7 and 75 mg was the dosage administered in 6/7 cases. Fever was the most common side effect present in 3/7 patients. Additionally, one patient developed low blood pressure and one patient limb pain and shivering. The most severe adverse event was a necrosis of the jaw, which occurred in 1/7 patients. Four patients did not report any adverse effects.

### 3.2. Patients Evaluate Bisphosphonate Treatment Positively

**Figure 1:**
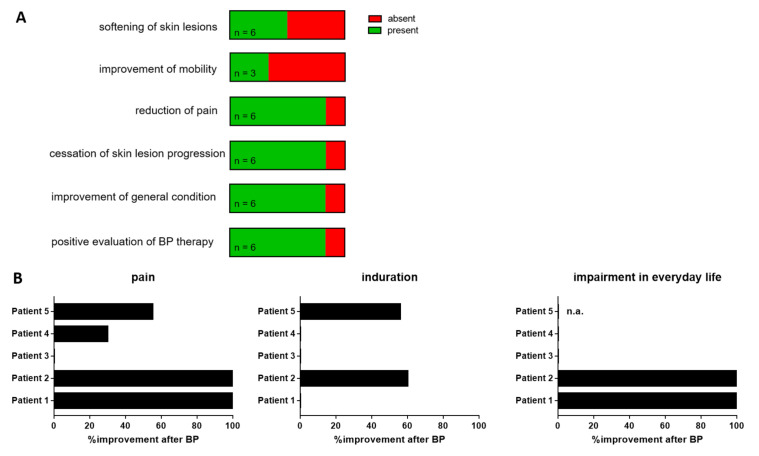
Patient’s subjective evaluation of bisphosphonate therapy effects. (**A**) Absence or presence of different treatment outcomes after initiation of bisphosphonate therapy as assessed by a postal questionnaire. (**B**) Percent improvement of pain, induration of calcinosis cutis skin lesions and impairment in everyday life after initiation of bisphosphonate therapy for each individual patient taking part in the questionnaire evaluation.

To assess patient satisfaction with bisphosphonate therapy, we asked five patients to estimate the effects on different aspects of CC (Figure 1) and analyzed the well-documented physician’s records of one deceased patient.

Three out of six patients noticed a softening of the CC skin lesions. Furthermore, 5/6 patients reported a reduction of pain, cessation of CC progression and improvement of their general condition. Three patients suffered from immobility of one or more joints before starting the bisphosphonate therapy. Of those, only one observed an improvement of mobility under treatment. Overall, 5/6 patients evaluated bisphosphonate therapy positively.

**Figure 2:**
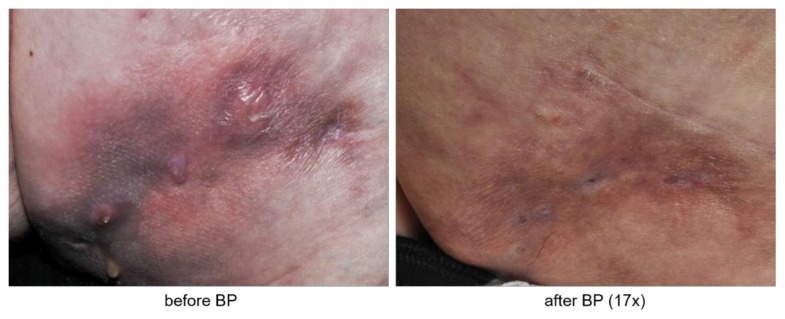
Representative clinical pictures of calcinosis cutis before and after bisphosphonate (BP) therapy (17 cycles).

Objective assessment reveals heterogeneous response to bisphosphonate therapy. While subjective perception is often biased by expected benefits, we evaluated clinical images and radiological reports before and after bisphosphonate therapy. Data were available for six patients. Here, we identified two patients with clear regression of CC skin lesions visible in clinical pictures after the initiation of bisphosphonate therapy (Figure 2).

**Figure 3:**
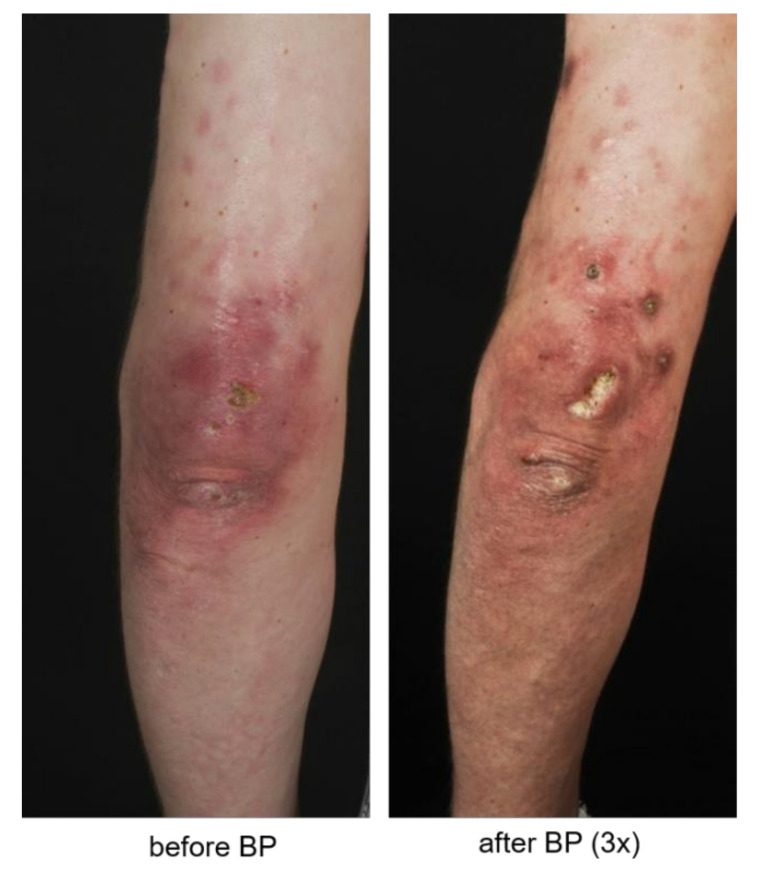
Representative clinical pictures of calcinosis cutis before and after bisphosphonate (BP) therapy.

One patient showed stable disease according to physician’s records. Three patients, however, developed additional or showed a progression of pre-existing CC lesions (Figure 3).

**Figure 4:**
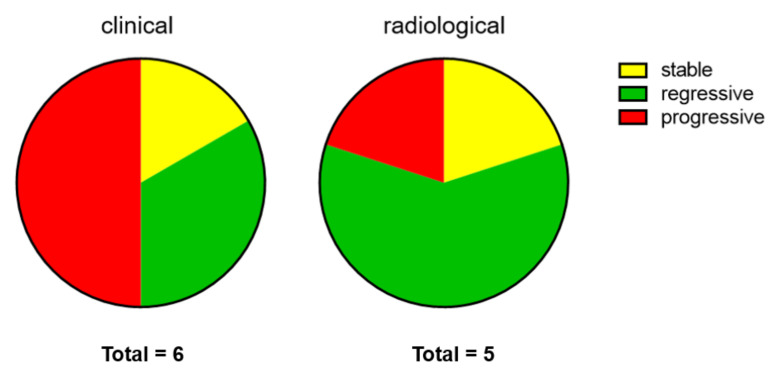
Objective evaluation of bisphosphonate therapy effects by dermatologists (clinical) and radiological examinations.

Radiological reports were available for 5/7 patients. The size of the CC was measured by conventional X-ray, computer tomography or magnetic resonance imaging. Here, improvement was reported in 3/5 patients, whereas 1/5 patients showed stable disease and 1/5 patients radiological progression. Concomitant treatment with prednisolone or methotrexate was not associated with a better outcome of CC. Thus, bisphosphonate treatment resulted in clinical and/or radiological improvement in approximately half of the patients, while 3/6 patients did not profit according to objective measurements (Figure 4).

## 4. Discussion

In this retrospective study we observed diverse clinical response to bisphosphonate therapy for the treatment of CC. While the vast majority of patients evaluated therapeutic effects positively, an objective improvement could only be observed in half of the patients. The most common side effect was fever. However, one patient developed osteonecrosis of the jaw, a known side effect of bisphosphonate treatment, which led to the discontinuation of therapy.

CC is a common symptom of autoimmune connective tissue diseases and one of the biggest therapeutic challenges. While negative effects on patient’s quality of life are immense, evidence for successful therapeutic concepts is rare. Dealing with different diseases, disease stages and heterogenous patient cohorts hampers the generation of homogenized and comparable patient cohorts and thereby the creation of evidence-based treatment guidelines [9]. Therefore, retrospective analysis of treatment outcome is a valuable tool to gain more insight into the therapeutic potential of different compounds.

Previous reports about bisphosphonates for CC are contradictory. Apart from positive results in single patient reports, the so far largest case series with six patients showed no beneficial effects of etidronate on CC [8]. Publication bias might account for an overestimation of therapeutical effects, as negative results are rarely published [10]. Our analysis includes all patients treated at our department within the last ten years. We found objective improvement or stabilization of CC in 4/7 patients. The diverse clinical outcome might be based on variance in disease duration, different mechanisms of CC formation or so far unknown individual factors contributing to CC development and maintenance.

The way that pamidronate influences CC is still speculative. Bisphosphonates decrease systemic calcium levels by inhibiting osteoclast activity. Thus, one potential mechanism of how pamidronate influences CC is an osmotic adjustment favouring resorption of calcium deposits from the subcutaneous tissue into blood vessels by lowering serum calcium levels. However, in contrast to metastatic calcification, CC in autoimmune diseases often develops independently from systemic shifts of calcium homeostasis [2]. Therefore, bisphosphonates might also have additional effects on CC lesions. Like many other mechanisms of autoimmune diseases, CC is thought to proceed in different stages. In the early stage, hypoxia or repeated local trauma triggers chronic inflammation at acral sites of the body. Destruction of connective tissue, tissue necrosis, immunological response to tissue degeneration and decreased activity of calcification inhibitors favour the accumulation of calcium depositions [11]. Often calcium deposits are accompanied by granulomatous inflammation comprising macrophages and foreign body giant cells. There is increasing evidence that bisphosphonate might also have anti-inflammatory effects on monocytes and macrophages [12,13]. Therefore, regression of CC under pamidronate therapy might be based on interrupting chronic inflammation. Given this potential mode of action, an analysis of the immunological signature accompanying CC might allow future stratification of patients. Patients with a high number of macrophages and monocytes due to foreign body reaction might be prone to better respond to bisphosphonate treatment than patients without inflammatory activity around CC lesions. Therefore, future studies should include histological and immunological evaluation of CC biopsies before the initiation of new therapeutic strategies.

Our study has limitations: First, we performed a retrospective study and not a randomized, placebo-controlled clinical trial. Therefore, there is no control group of untreated patients or patients receiving a placebo. Second, as we summarized data from clinical routines, the time between the first pamidronate infusion and radiological assessment varies between different patients. Third, there is a potential recall bias as patients filled out the questionnaire years after the last pamidronate cycle. However, as CC is a very difficult-to-treat symptom of connective tissue diseases with a high unmet medical need for new therapeutic options, this comprehensive retrospective analysis including subjective and objective outcome parameters is of high value for clinicians treating patients with CC.

In summary our study demonstrates that a subgroup of CC patients might profit from bisphosphonate treatment. However, as parameters predicting therapeutic response are still missing and severe adverse events can occur, treatment with bisphosphonates has to be evaluated carefully. Assessment of treatment outcomes should be based on objective findings rather than on a patient’s subjective perception, which is biased by individual expectations. Future studies better investigating the exact mechanisms of CC formation in autoimmune diseases will allow better identification of patient subgroups with a high probability to respond well to bisphosphonate treatment.

## Figures and Tables

**Table 1:** Patients’ characteristics.

Participant ID	1	2	3	4	5	6	7
sex	male	female	female	female	female	female	female
diagnosis	dermato-myositis	dermato-myositis	mixed connective tissue disease	mixed connective tissue disease	systemic sclerosis	mixed connective tissue disease	systemic sclerosis
age at disease onset (years)	26	46	48	10	58	47	38
age at onset of calcinosis cutis (years)	26	unknown	50	14	59	49	unknown
time between beginning of calcinosis cutis and initiation of bisphospho-nate treatment (years)	8	unknown	8	13	4	2	unknown
number of bisphospho-nate cycles	7	12	3	6	12	18	2
dosage (mg)	70	75	75	75	75	75	75
previous treatments	intravenous immuno-globulins cyclophosphamid methyl-prednisolone azathioprine	methotrexate prednisolone azathioprine	intravenous immuno-globuline methotrexate prednisolone azathioprine chloroquine PUVA, iloprost	methotrexate prednisolone cyclosporine chloroquine	bosentan pentoxi-fylline	hydroxy-chloroquine azathioprin mycophenolate mofetil intravenous immuno-globulins prednisolone iloprost	prednisolone
treatment during bisphospho-nate therapy	methyl-prednisolone	methotrexate prednisolone	intravenous immuno-globulines methotrexate prednisolone	methotrexate prednisolone	pentoxi-fylline	-	prednisolone
adverse events	-	fever low blood pressure	-	fever shivering limb pain	fever osteonecrosis of the jaw	-	-

## Data Availability

The authors confirm that the data supporting the findings of this study are available within the article and its Supplementary Materials.

## Supplementary Materials

The following are available online at https://www.mdpi.com/article/10.3390/biomedicines9111698/s1. Questionnaire for the research project: Disodium pamidronate for the treatment of calcinosis cutis–a retrospective single-center study.
