# Supplementary material for: Bisphosphonates for the Treatment of Calcinosis Cutis—A Retrospective Single-Center Study
Source: Biomedicines. 2021 Nov 16;9(11):1698. doi: 10.3390/biomedicines9111698 (PMC8615716; doi:10.3390/biomedicines9111698)
Supplement: Supplementary file 1 [file biomedicines-09-01698-s001.zip › biomedicines-1419877-supplementary.pdf]

Questionnaire for the research project: Disodium pamidronate for the treatment of calcinosis cutis–a retrospective single-center study.

Study director: Felix Lauffer, Doctor of Medicine; assistant medical director: Lilian Rauch, medical student.

(1) What is the underlying disease that leads to calcium deposits in your skin?

(2) How old were you or what year was it when the diagnosis was made by a doctor?

(3) What year was it or how old were you when you first noticed induration or calcification of the skin?

(4) On which parts of your body did you first notice the induration of the skin or calcifications?

(5) (a) Was the calcified skin painful?

Yes ☐ No ☐

(b) If so, when did you first experience pain in the calcified areas?

(6) Which treatments, at home and in the hospital, (pills, syringes, infusions, et al.) had you received before the first treatment with bisphosphonates? In which period? Was it effective against calcification of the skin? Please fill out the following table.

Name of the treatment: Period of administration: Effective against calcification?

(7) When did you get the first infusion of bisphosphonates?

(8) Was the first infusion administered in the Department of Dermatology and Allergy, Technical University of Munich?

Yes ☐ No ☐

(9) How many cycles of bisphosphonate infusions did you receive in total (a cycle consists of three infusions on three consecutive days)?

amount: cycles

(10) Why was the treatment not continued?

(11) (a) Is it planned to restart the treatment?

Yes ☐ No ☐

(b) Why/Why not?

(12) (a) Did any side effects occur? If so, which ones and in which period?

(b) Have you received any medication or treatment because of the side effects?

Yes ☐ No ☐

(c) If so, which treatment did you receive?

(d) Did you feel strongly affected by the side effects?

Yes ☐ No ☐

Please explain why.

(13) The time interval between bisphosphonate cycles is usually around 3 months.

Could you notice any aggravation at the end of the 3 months?

Yes ☐ No ☐

If so, how could you notice it?

(14) (a) Which part of the body was most affected by calcification before the first bisphosphonate infusion?

(b) How severe was the induration on that part of your body (mentioned at 14a) before the first bisphosphonate infusion? Please indicate the grade of induration on a scale from 0–10. The number 0 means no induration, the number 10 maximum induration.

(c) Was that part of your body (mentioned at 14a) painful? If so, how severe was the pain before the first bisphosphonate infusion? Please indicate the grade of induration on a scale from 0–10. The number 0 means no pain, the number 10 stands for the most intense pain you can imagine.
(d) How severe was the induration on that part of your body (mentioned at 14a) after the end of the last cycle of bisphosphonate infusions? Please indicate the grade of induration on a scale from 0–10. The number 0 means no induration, the number 10 maximum induration.
(e) On a scale from 0–10, how severe was the pain at that part of your body (mentioned at 14a) after the end of the last cycle of bisphosphonate infusions? The number 0 means no pain, the number 10 stands for the most intense pain you can imagine.
(f) How is the skin on that part of your body (mentioned at 14a) today? How severe is the induration? Please indicate the grade of induration on a scale from 0–10. The number 0 means no induration, the number 10 maximum induration.
(g) Is that part of your body (mentioned in 14a) currently painful? If so, how severe is the pain on a scale from 0–10? The number 0 means no pain, the number 10 stands for the most intense pain you can imagine.
(15) (a) Did you have limitations of mobility caused by calcification before the first bisphosphonate infusion? If so, which part of the body was the most limited of mobility?
(b) On a scale from 0–10, how severe was the limitation of mobility before the first bisphosphonate infusion? The number 0 means no limitation of mobility, the number 10 stands for complete limitation of mobility/movement almost impossible.
(c) On a scale from 0–10, how severe was the limitation of mobility after the last cycle of bisphosphonate infusions? The number 0 means no limitation of mobility, the number 10 stands for complete limitation of mobility/movement almost impossible.
(d) On a scale from 0–10, how severe is the limitation of mobility today? The number 0 means no limitation of mobility, the number 10 stands for complete limitation of mobility/movement almost impossible.
(16) Did you feel impaired in everyday life by the calcification of your skin? The number 0 means not impaired, the number 10 stands for the most severe impairment you can imagine. Grade of impairment before the first infusion (0–10):
Grade of impairment after the end of the last cycle of bisphosphonate infusions (0–10):
(17) Overall, do you have the impression that the treatment with bisphosphonates had a positive effect on you?
Yes ☐ No ☐ Partially ☐
If "partially", please explain:
(18) Did the treatment with bisphosphonates lead to a reduction of pain?
Yes ☐ No ☐ Partially ☐
If "partially", please explain:
(19) Did the progress of calcification slow down?
Yes ☐ No ☐ Partially ☐
If "partially", please explain:
(20) Did the indurated skin become softer?
Yes ☐ No ☐ Partially ☐
If "partially", please explain:

(21) Did you notice an improvement in mobility?

Yes ☐ No ☐ Partially ☐

If "partially", please explain:

(22) Did the treatment with bisphosphonates have a positive effect on your general condition?

Yes ☐ No ☐ Partially ☐

If "partially", please explain:

(23) Have existing calcifications of the skin become worse since the end of the treatment with bisphosphonates?

Yes ☐ No ☐ Partially ☐

If "partially", please explain:

(24) (a) Have new calcified lesions appeared on your skin since the end of the treatment with bisphosphonates?

Yes ☐ No ☐

(b) If so, how many new calcified lesions have appeared?

(c) If so, on which parts of your body have they appeared?

(25) Has pain increased since the end of the treatment with bisphosphonates?

Yes ☐ No ☐ Partially ☐

If "partially", please explain:

(26) Have limitations of mobility become worse since the end of the treatment with bisphosphonates?

Yes ☐ No ☐ Partially ☐

If "partially", please explain:

(27) Which factor do you consider most important or decisive in the evaluation of the treatment with bisphosphonates?

Free page for possible additions:
